# KIN NETWORK DYNAMICS OVER THE LIFE COURSE AND HISTORICAL TIME IN 19TH-CENTURY ORKNEY, SCOTLAND

**DOI:** 10.1093/geroni/igz038.622

**Published:** 2019-11-08

**Authors:** Julia Jennings

**Affiliations:** University at Albany, Albany, New York, United States

## Abstract

Kin are important sources of social, instrumental, and financial assistance for older adults. Support from kin is associated with improved wellbeing and longer lives among this age group, yet few longitudinal studies examine information on the composition and structure of kin networks beyond dyadic relationships, such as those between spouses or parents and their children. This study examines the dynamics of non-dyadic measures of kin networks among adults over age 60 using multiple longitudinal linked data sources from North Orkney, Scotland, 1851-1911. Reconstructed individual life courses (N=4,946) and genealogies, in combination in spatial information concerning the proximity non-coresident kin, are used to examine change in kin availability and propinquity over the life course and across historical time. Orkney provides an interesting case study; as information is available on individual-level change in kin availability with a long period of follow up during a time of population change. The study period covers the early stages of population aging and depopulation of the islands, which began in the 1870s in this community. A descriptive analysis of kin network change is presented. Kin availability is associated with longer lives in this sample. The presence of co-resident kin is associated with economic status, after controlling for other factors. Older adults who receive poor relief are significantly more likely to live alone and less likely to live with kin, and the association is stronger for men than for women.

